# Quantitative Monitoring of Tattoo Contrast Variations after 755-nm Laser Treatments in In Vivo Tattoo Models

**DOI:** 10.3390/s20010285

**Published:** 2020-01-04

**Authors:** Myeongjin Kim, Suhyun Park, Hyun Uk Lee, Hyun Wook Kang

**Affiliations:** 1 Interdisciplinary Program of Marine-Bio, Electrical and Mechanical Engineering, Pukyong National University, Busan 48513, Korea; mjkim@pukyong.ac.kr; 2 School of Electrical and Electronics Engineering, Chung-Ang University, Seoul 06974, Korea; suhyun@cau.ac.kr; 3 Bluecore Company Co., Ltd., Busan 48059, Korea; bluecore.lhu7325@gmail.com; 4 Department of Biomedical Engineering and Center for Marine-Integrated Biomedical Technology (BK21 Plus), Pukyong National University, Busan 48513, Korea

**Keywords:** CMOS sensor, image contrast, laser treatment, tattoo model

## Abstract

Laser lights have been used by dermatologists for tattoo removal through photothermal interactions. However, most clinical studies used a visual scoring method to evaluate the tattoo removal process less objectively, leading to unnecessary treatments. This study aimed to develop a simple and quantitative imaging method to monitor the degree of tattoo removal in in vivo skin models. Sprague Dawley rat models were tattooed with four different concentrations of black inks. Laser treatment was performed weekly on the tattoos using a wavelength of 755 nm over six weeks. Images of non-treated and treated samples were captured using the same method after each treatment. The intensities of the tattoos were measured to estimate the contrast for quantitative comparison. The results demonstrated that the proposed monitoring method quantified the variations in tattoo contrast after the laser treatment. Histological analysis validated the significant removal of tattoo inks, no thermal injury to adjacent tissue, and uniform remodeling of epidermal and dermal layers after multiple treatments. This study demonstrated the potential of the quantitative monitoring technique in assessing the degree of clearance level objectively during laser treatments in clinics.

## 1. Introduction

Tattoos have played an important role in diverse human cultures [1]. They are a form of body art, wherein ink is injected into the dermis of the skin. When ink is inserted into the dermis, the immune system that recognizes the ink molecules as foreign substances is triggered to recruit macrophages. However, the inks remain, because the lysosomal enzymes are still inactive in the ink components [2,3]. Individuals with tattoos sometimes seek the removal of tattoos because of various reasons, such as changes in social status, dissatisfaction, and medical problems [4]. Consequently, a proficient tattoo removal process is required. Various tattoo removal methods include surgical excision and chemical destruction. However, laser tattoo removal is currently considered to be one of the most efficient techniques owing to its selective treatment. The laser approach targets certain chromophores and has minimal interaction with the adjacent tissue, thereby making it more specific, less invasive, and associated with fewer side effects [5]. Several laser wavelengths have been evaluated to demonstrate their clinical outcomes [6,7,8]. For example, the CO_2_ laser (10.6 μm) uses low light absorption for the tattoo pigments, which causes unwanted fibrotic scars after treatment [6]. In contrast, visible (532 nm) or near-infrared lasers (755 and 1064 nm) can destroy pigment particles selectively with short and intense laser pulses through selective photothermolysis [7]. In particular, the wavelengths of 755 and 1064 nm have shown stimulations in inflammatory responses that can eliminate the fragmented tattoo granules through lymphatics [8].

Several research works have been widely conducted on laser tattoo removal under preclinical and clinical conditions [9,10,11,12,13,14]. Typically, laser removal progresses through several treatment sessions to achieve the complete removal of the tattoo ink. However, previous studies poorly understood the quantitative information on the amount of residual tattoo ink after each treatment. Dermatologists generally use a scoring system to measure the clearance of tattoos after the laser treatment [11,12,13,14]. The scoring system is based on a clinical scale of poor (<25% removal), fair (26%–50% removal), good (51%–75% removal), and excellent (76%–100% removal). However, the score is analyzed subjectively by medical experts, and is hardly a quantitative indicator to present the amount of tattoo ink removal, thereby leading to unnecessary treatment sessions. Moreover, the side effects of laser tattoo treatment include fibrotic scars, hypo- or hyper-pigmentation, and tattoo pigment color changes due to the incomplete removal or excessive/accumulated thermal damage after multiple treatment sessions [15,16]. Therefore, it is necessary to establish a reliable and objective method to assess the degree of tattoo removal after each laser treatment session.

A quantitative monitoring method was developed in this study to measure the temporal variations in tattoo contrast after 755 nm laser treatments in in vivo tattoo models. It was hypothesized that the assessment of image contrast between pre- and post-treatments could reliably quantify the degree of clearance level in the tattoos after each treatment. Multiple 755 nm laser treatments were conducted in vivo on skin tattoos with four different concentrations. A complementary metal-oxide-semiconductor (CMOS) sensor was used to monitor each treated skin tattoo and the acquired images were evaluated in terms of tattoo contrast over six weeks. Histology analysis was also performed to validate the presence of tattoo ink under the epidermis and to assess any thermal responses in the treated tissue and post-treatment wound healing.

## 2. Materials and Methods

### 2.1. Animal Preparation

Animal experiments were approved by the Institutional Animal Care and Use Committee at Pukyong National University (Number 2018-22). A total of eight male Sprague Dawley rats (aged six weeks and weighing 150–200 g) were used as the animal models for this study. Before the experiments, all of the animals inhaled isoflurane (Terrell™ isoflurane, Piramal Critical Care, Bethlehem, PA, USA) for anesthesia. The back of each rat was shaved with a clipper and the hair was completely removed using a waxing cream. The tattoos were made using a reciprocating vibrator-driven needle machine and black ink (Crazy black, D&H Company, Incheon, Korea) that was injected into the skin less than 1 mm below the epidermis. Eight 1.5 cm long tattoo lines (1 cm apart; ~1.5 mm thick) were created on the back of each rat at various concentrations. To reflect clinical tattoo conditions (fresh vs. faint), the current study tested four different concentrations (i.e., 25%, 50%, 75%, and 100%) by diluting the ink with a solution (Tattoo Ink Solution, D&H Company, Incheon, Korea). Each type of tattoo ink was injected four times (every three days) to completely establish a tattoo line on the skin. Thus, the laser treatments were performed after the tattoos were adequately engraved (i.e., 12 days in total). The tattoo lines of each concentration were created on the skin for two groups, namely: control (non-treated) and laser-treated groups. A total of 64 samples was prepared (i.e., number of concentrations × number of lines × number of groups = 4 × 8 × 2 = 64).

### 2.2. Laser Treatment

**Figure 1:**
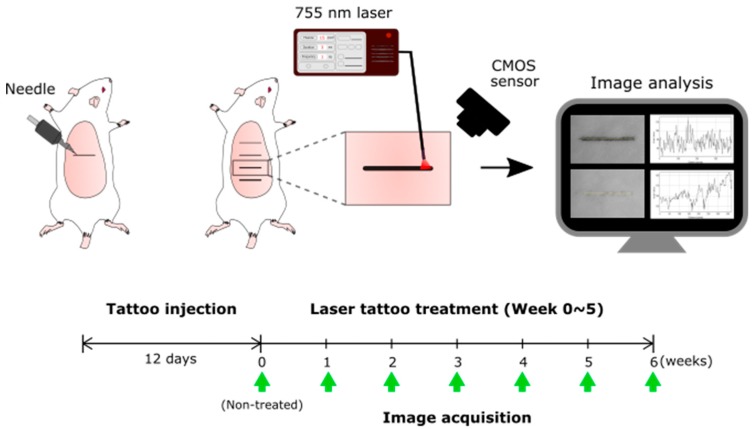
Experimental setup to quantitatively monitor the laser tattoo treatment. Green arrows represent the specific times for image acquisition. Note that each image was acquired one week after each laser treatment (CMOS: complementary metal-oxide-semiconductor).

A customized long-pulsed 755 nm laser system (pulse duration = 3 ms; Bluecore Company, Busan, Korea) was used as the light source for the tattoo treatment. The laser system was operated with a pulse energy of 5 J, frequency of 1 Hz, and beam diameter of 6.5 mm in order to emulate the clinical treatment conditions [17]. The corresponding fluence was 15 J/cm^2^, and each tattoo line was manually treated with five pulses of the 755 nm laser irradiation. After every five pulses, the beam was moved by 5 mm laterally until it covered the entire line (i.e., 15 pulses per tattoo line). Figure 1 displays an experimental setup for quantitatively monitoring the laser treatment that was performed on each tattoo concentration once a week for six weeks (six treatments in total; week 0–5 after imaging in Figure 1), because our preliminary testing confirmed that no further tattoo removal was required after the six weeks of treatments. Photographs were taken on non-treated (week 0) and treated tattoos per week after each treatment (week 1–6; green arrows in Figure 1) using a CMOS sensor (DMK AFUX236-M12, Imaging Source, Taipei City, Taiwan) to monitor the collective responses in laser-induced contrast variations and the tissue healing processes. The sensor captured 2464 × 1632 images with a pixel size of 2.8 × 2.8 μm. As the proposed method envisioned an application under community settings for easy acceptance and set-ups, each image was acquired under the illumination of incandescent light (60 W and 800 lumens), and no imaging filter was applied. For consistent acquisition for post-experimental analysis, imaging conditions (intensity of illumination light source, 10-cm vertical distance between image and object planes, and no environmental light) were maintained constantly.

### 2.3. Contrast Analysis

A week after each weekly treatment, the laser-treated samples were photographed for contrast measurements. Image acquisition and analysis were performed to reflect the collective degree of clearance level after physical removal and the ensuing wound healing resulting from the treatment of the previous week. Once the image was captured, the next treatment was performed. Five different sites were randomly selected from each tattoo line, and the corresponding intensity in grayscale was measured using the Image J software (National Institute of Health, Bethesda, MD). The non-tattooed area was used as the background, and its intensity was also quantified for reference purposes. To minimize measurement errors from the acquired images, the contrast change (C) between the background and the treated site was calculated using the following equation:

Contrast change (C) = (I_B_ − I_T_)/I_B_ × 100 (1)

where I_B_ and I_T_ are the intensities measured from the background and tattoo, respectively. The contrast estimated after laser treatment (week 1–6; Figure 1) was compared to that estimated before the treatment (i.e., non-treated; week 0; Figure 1) to quantify the degree of tattoo removal after the laser treatment.

### 2.4. Histology

A histological analysis was performed to confirm the spatial distribution of the ink particles in both non-treated and treated skin tattoos, accumulated thermal injury, and the wound healing responses of the tattoo after multiple treatments for six weeks. Once the six laser treatments were completed, all of the animals were euthanized and the skin tissue was harvested. The extracted samples were fixed in 10% formalin for 48 h. After tissue processing, the samples were embedded in paraffin and sectioned by 6-μm thickness. Then, all of the sectioned tissues were stained with hematoxylin and eosin (HE), and the stained slides were observed using an optical microscope to acquire images (magnification = ×100). To quantitatively evaluate the extent of the epidermal variations between the normal and treated tissues, their epidermal thickness index (ETI) was calculated by normalizing the epidermal thickness measured from the treated tissues by that from the normal tissue.

### 2.5. Statistical Analysis

All of the acquired data were represented as mean ± standard deviation. A Mann–Whitney U-test was conducted for the non-parametric statistical analysis, where *p* < 0.05 expresses the statistical significance of the data sample.

## 3. Results

**Figure 2:**
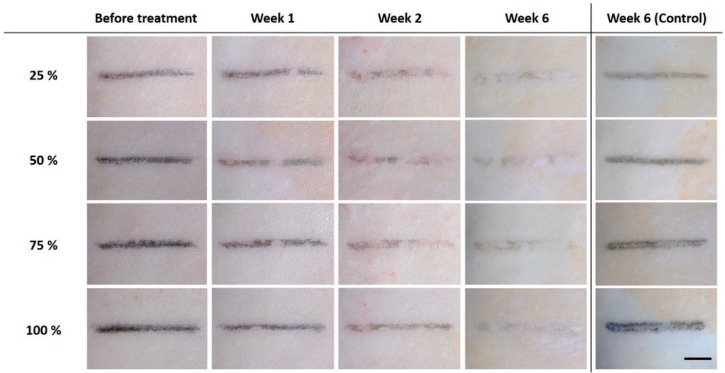
Top surface images of tattoo on skin before (week 0) and after weekly laser treatment (weeks 1, 2, and 6). The far-right column shows images of non-treated tattoo surfaces acquired at week 6 (control; scale bar = 5 mm).

Figure 2 displays the top surface images of the skin tattoo lines with four different ink concentrations at various stages of post treatment. The before treatment stage (week 0; far left column) showed that the higher concentrations indicated more distinctive tattoo lines. For instance, 100% was associated with a relatively dark black line, whereas 25% with a slightly faint one. Irrespective of the ink concentrations, weekly laser treatment continued to induce tangible reductions in the amount of residual tattoo with time. At week 6, the lower concentrations (25% and 50%) resulted in a significant removal of the tattoos (almost indiscernible), while the highest concentration (100%) still left a relatively distinguishable tattoo line. It was confirmed that the control group (non-treated; week 6) maintained a comparable degree of tattoo concentration over four weeks (far right column in Figure 2) to that from week 0 (before treatment).

**Figure 3:**
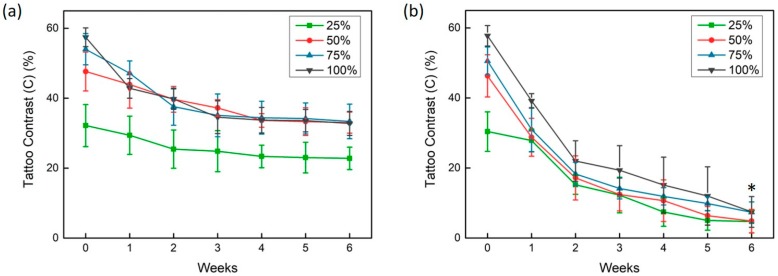
Variations in tattoo contrast as a function of time for four concentrations, namely: (**a**) control (non-treated) and (**b**) laser-treated tattoo samples (n = 5 per concentration; * *p* < 0.05 vs. week 0). Note that week 0 in (**b**) represents the tattoo contrast measured before laser treatment, and weeks 1–6 represents the contrast measured one week after each treatment.

Tattoo contrast was quantified as a function of time for non-treated and treated groups with four concentrations. According to Figure 3a, the control (non-treated) group showed that three concentrations (50%, 75%, and 100%) yielded a contrast reduction of up to 25% after six weeks (week 0: 57.4 ± 2.7% vs. week 6: 32.8 ± 3.4% for 100%; *p* < 0.05). The lowest concentration (25%) reduced the contrast by approximately 9% (week 0: 32.2 ± 6.0% vs. week 6: 22.8 ± 3.2% for 25%; *p* < 0.05). Despite the lower contrast, the overall contrast changes for the 25% concentration had a comparable tendency to those from the other concentrations. Thus, it was confirmed that all of the control groups retained tattoo ink in the skin almost invariably over six weeks (Figure 3a). In contrast, Figure 3b demonstrates that the treated groups indicated a distinctive and rapid reduction in tattoo contrast (up to 49%) with multiple treatments for all of the concentrations (week 0: 57.8 ± 2.9% vs. week 6: 9.0 ± 6.6% for 100%; *p* < 0.05). The degree of weekly contrast reduction was significantly similar for all of the treated groups. Quantitative monitoring of the continuous contrast reduction in the treated groups indicated that laser treatment could remove more tattoo ink.

**Figure 4:**
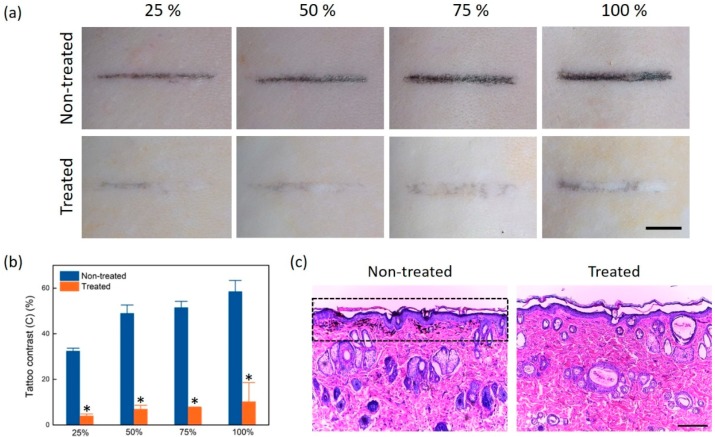
Comparison of non-treated (week 0) and laser-treated tattoo samples (week 6): (**a**) top surface images (scale bar = 5 mm), (**b**) tattoo contrast for four different concentrations measured from Figure 4a (* *p* < 0.05 vs. non-treated), and (**c**) histology images of non-treated (week 0) and treated skin tattoo (concentration = 75%; week 6). Note that the dashed lines represent the ink particles injected into the dermis of non-treated tissue (scale bar = 200 μm; × 100).

Figure 4 compares the tattoo lines with four different ink concentrations between the non-treated (week 0) and laser-treated groups (week 6). According to Figure 4a, all of the concentrations yielded distinctive tattoo lines on the skin. After six weeks of treatment, the captured images confirmed that all of the tattoo lines became evidently faint. The degree of residual tattoo was gradually more palpable with higher concentrations. Figure 4b demonstrates a comparison of tattoo contrast between the non-treated (week 0) and laser-treated groups (week 6). The tattoo contrast increased with the ink concentration for both of the groups. Irrespective of the concentrations, the tattoo contrast was substantially reduced by a factor of up to six after the laser treatment (*p* < 0.05 for all of the concentrations). Figure 4c demonstrates the histology images of a tattoo line (concentration = 75%) from the non-treated (week 0; left) and the treated groups (week 6; right). The dashed box in the non-treated image in Figure 4c qualitatively confirmed the existence of injected tattoo (black) ink in the dermal layer. After the six treatments, the treated groups validated the evident disappearance of the injected ink particles from the skin.

**Figure 5:**
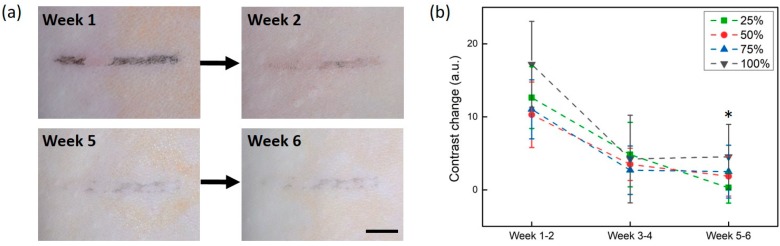
Reductions in tattoo contrast after weekly laser treatment: (**a**) top surface images after the first and second treatments (top; weeks 1 and 2) and fifth and sixth treatments (bottom; weeks 5 and 6; concentration = 50%), and (**b**) variations in contrast changes between two consecutive weeks for four different concentrations (scale bar = 5 mm; * *p* < 0.05 vs. weeks 1–2).

Figure 5 presents the degree of tattoo removal between two consecutive weeks. Week 1 (first treatment) showed a partially removed tattoo line, whereas the next treatment (week 2) led to a considerably removed tattoo line (concentration = 50%; top images in Figure 5a). In contrast, both week 5 (bottom left image in Figure 5a) and week 6 (bottom left image in Figure 5a) resulted in relatively invisible tattoo lines, and yielded almost indiscernible changes in the tattoos after consecutive treatments. Figure 5b quantifies the changes in the tattoo contrast measured for three different consecutive periods (weeks 1–2, weeks 3–4, and weeks 5–6). For all of the concentrations, the earlier treatments (weeks 1–2) induced marked tattoo contrast changes (up to 17%). However, the later treatments (weeks 3–4 and weeks 5–6) accompanied similar contrast changes (around 5%). Larger variations at each period resulted from a partial removal of the tattoo line due to an uneven accumulation of ink molecules and the resultant non-uniform laser interactions. This monitoring method verified that the tattoo removal efficiency could substantially decrease when the frequency of laser treatment is increased.

**Figure 6:**
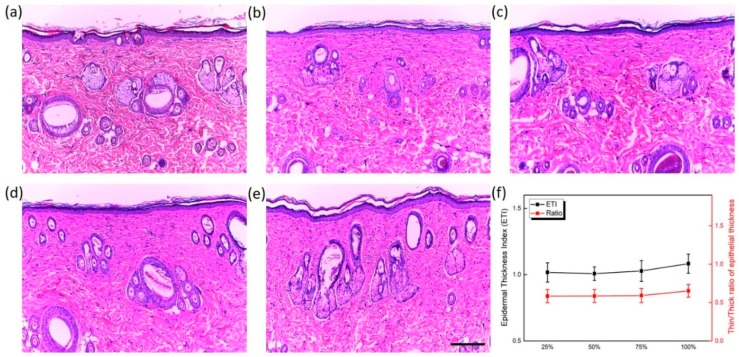
Hematoxylin and eosin (HE) staining images acquired after six laser treatments for various concentrations (week 6; scale bar = 200 μm): (**a**) normal tissue (non-injected and non-treated), (**b**) 25%, (**c**) 50%, (**d**) 75%, (**e**) 100%, and (**f**) quantitative comparison of epidermal thickness index (ETI) and thin/thick ratio in epidermis (10 measurements per concentration).

Figure 6 displays the histology images of the normal tissue (Figure 6a) and laser-treated tissues with various concentrations (week 6; Figure 6b–e) to evaluate the degree of thermal damage and wound healing after six laser treatments. Despite the six weekly treatments, no thermal injury was qualitatively found in the epidermis and dermis for all of the concentrations. It was noted that the remodeled skin structures after the treatments seemed equivalent to the structure of the normal tissue, irrespective of the concentrations. Figure 6f exhibits the epidermal thickness index (ETI) and ratios of thin/thick epidermal thicknesses. Evidently, the treated tissues remodeled the thicknesses of the epidermis comparable to that of the normal tissue, even after the six laser treatments (ETI = 1.0−1.1). The quantified thickness ratios also confirmed that the remodeling of the epidermal layers was significantly uniform and no accumulated thermal injury was observed in all of the concentrations.

## 4. Discussion

This study aimed to develop an objective and quantifiable method to monitor the variations in tattoo contrast after 755 nm laser treatment in in vivo skin models. Unlike the current scoring system used for clinical evaluations, the proposed monitoring method presented substantial reductions in tattoo contrast after the weekly laser treatments in a qualitative and quantitative manner (Figure 2, Figure 3 and Figure 4). Because subjective visual inspections of tattoo treatments often lead to unnecessary treatment sessions, establishing an objective and quantitative approach to evaluate the degree of tattoo removal is a clinical unmet need. The proposed imaging analysis can enable monitoring the variations in tattoos quantitatively in clinics. Furthermore, implementation of the proposed method with smartphones or low-cost alternative devices can facilitate clinical capability and acceptance by medical communities. Continuous imaging with such implementations can even provide additional benefits to monitoring the degree of tattoo removal. The weekly contrast changes evidently showed the amount of variations in the clearance level for each treatment (Figure 5), which can be used to define the optimal number of laser treatments so as to avoid any unnecessary treatments. Histological analysis confirmed the significant removal of tattoo ink, no thermal injury to the adjacent tissue, and uniform remodeling of epidermal/dermal layers after multiple treatments (Figure 6). Therefore, the proposed monitoring technique can be a non-invasive method to evaluate the physical status of skin after laser treatment in a rapid and quantifiable manner, and eventually, improve the clinical outcomes of laser tattoo removal by minimizing the number of treatments.

The ultimate goal of tattoo laser removal is the fragmentation of tattoo ink particles that are easily removed by the host immune system from the skin. The main mechanism of laser tattoo interactions is considered to be photothermal, and the degree of tattoo removal may also affect the properties of pigment molecules [16]. This study sought to quantify the physical responses of tattoo inks with various concentrations to pulsed laser irradiation. It was assumed that in the ink with a higher concentration, more particles were removed on account of more light absorption by the tattoo chromophores. However, the overall tattoo contrast was lower for lower concentrations instead of higher concentrations (Figure 3b). These findings implicate that the applied treatment dosage may be effective for the removal of a certain degree of tattoo ink, owing to the various sizes and spatial distributions of the injected particles. The resultant interactions of the chromophores with laser light typically accompany partial light absorption and scattering of the incident light, thereby leading to the re-distribution of the particles [18]. Further studies will be pursued in order to evaluate the optical and thermal effects of tattoo particles on the efficiency of laser tattoo treatment in terms of size, density, spatial distribution, and dependence on a number of treatments. In addition, the concentration-dependent treatment dosage will be investigated using the proposed method to define the optimal treatment planning.

Clinical research on tattoo treatment has used clinical scoring rates to identify any reduction in treated tattoos [5,9,11,12,13,14,19,20]. However, clinical assessment could be subjective and non-quantitative, because the scores are given by medical experts or blinded researchers. As an alternative method, quantitative evaluations using animal models were developed [5,9,20]. A study evaluating the laser effect on Hartley guinea pigs with multi-colored tattoos used a colorimeter to measure any changes in pigmentation [9]. However, the calculation of absorbance was still complex, and the method merely showed the total reduction between pre- and post-treatment, whereas the proposed method could monitor the variations in tattoo contrast, even during wound healing. Another study using rat models reported the application of a skin assessment instrument to determine the pigment contents before and after the laser treatment and to estimate the clearance rate [20]. However, the quantitative measurement required a process wherein the instrument was tightly applied to the animal skin in a vertical direction. Moreover, the intensities of black points from an image of the tattooed area was quantified to assess the efficiency of laser treatment using a color threshold adjustment tool in Image J [5]. However, the process of threshold adjustments was often subjective (measurer-dependent). Several iterations were also essential for minimizing measurement errors and achieving assessment reliability. The formula to determine the clearance rate was more complicated and less accurate than the proposed method, with a direct comparison of intensities between background and treated regions. Unlike a colorimeter and skin assessment instrument, a CMOS-based sensor can provide a simple and cost-effective set-up for consistent imaging with high accuracy. Therefore, this research demonstrated a simple method of using 2D images with a CMOS sensor to monitor the amount of reduction per laser treatment in a reliable and quantitative manner.

Although this study showed a quantitative monitoring method using a CMOS sensor to assess the degree of tattoo removal, experimental limitations still remain. The proposed long-pulsed (3 ms) laser yielded no accumulated thermal injury, as shown in Figure 6, but longer pulse durations may damage the surrounding tissue, unless thermal confinement is satisfied (i.e., pulse duration longer than the thermal relaxation time of 3–10 ms) [18]. Moreover, this study measured the intensities of the tattoo lines in grayscale, because only black tattoo inks were used. However, it is important to understand whether the monitoring method can be applicable for various tattoo ink colors, such as red, green, and blue. Thus, further investigations will apply the proposed monitoring method to laser treatments with short pulse durations to validate the current findings in color tattoo models. Additionally, the efficiency of particle fragmentation with laser light (i.e., lower therapeutic effect) is dependent on the depth of pigment particles in skin. To ensure a uniform distribution of tattoo ink, a method to monitor the depth of the tattooed skin needs to be developed prior to the laser treatment. Despite the verification of the initial existence and the complete removal of tattoo molecules, histology images could hardly visualize the partial presence of the residual ink molecules, possibly due to reduced/scattered ink distribution, limited image resolution, and relatively imprecise tissue sectioning (Figure 4c). The illuminating light condition can be another critical factor for image acquisitions and intensity measurements with Image J. Image acquisition should be performed without changes in external conditions, including ambient lighting for clinical translation. In addition, the limitation should be minimized by measuring the intensities from the background and tattoo simultaneously. The eventual goal is to quantify the contrast changes in real-time, and determine the optimal number of laser treatments. Therefore, the optimal (or minimal) number of treatments may lead to a reduced cost of clinical tattoo treatment, as well as any treatment-associated pain that patients can experience.

## 5. Conclusions

In this study, we developed a quantitative monitoring method for tattoo contrast variations after 755 nm laser treatment in in vivo skin models. The proposed monitoring method qualitatively and quantitatively assessed the tattoo contrast and confirmed no thermal damage after multiple treatments. The favorable findings of the proposed method will further be evaluated using laser systems with shorter pulse durations in color tattoo models so as to optimize the number of treatments for clinical translation.
